# Innovations in Photogrammetry and Remote Sensing: Modern Sensors, New Processing Strategies and Frontiers in Applications

**DOI:** 10.3390/s21072420

**Published:** 2021-04-01

**Authors:** Francesco Mancini, Francesco Pirotti

**Affiliations:** 1 Department of Engineering ‘Enzo Ferrari’, University of Modena and Reggio Emilia, 41125 Modena, Italy; 2 CIRGEO Interdepartmental Research Center of Geomatics, TESAF Department, University of Padua, 16035 Legnaro, Italy; francesco.pirotti@unipd.it

The recent development and rapid evolution of modern sensors and new processing strategies of collected data have paved the way for innovations in photogrammetry and remote sensing. The observation of the natural and built environment from aerial and satellite platforms benefits from improvements in data processing strategies thanks to the introduction of approaches based on multi-sensor data fusion, near-real-time mapping and monitoring, the introduction of a new generation of algorithms from artificial intelligence for data processing, and increased ability in the handling of a big dataset. The new generation of sensors and methods in the fields of photogrammetry and remote sensing has also been complemented by an ever-greater level of automation and capabilities in data processing. A wide range of professional and research activities, previously limited to geomatics engineering and Earth observation, now have the opportunity to exploit the aforementioned tools in the timely investigation of natural or anthropogenic features and phenomena. More flexible and automated approaches are also required by markets related to the space economies promoted by several countries around the world, and the new opportunities arising from the use of sensors designed for photo- or video-monitoring applications from low-altitude unmanned aerial vehicles (UAVs) or fixed locations in terrestrial surveys. In this direction, much has been achieved by the professionals and researchers in terms of the accurate reconstruction of three-dimensional features at very high spatial and temporal resolutions using close-range photogrammetry and structure from motion (SfM) technology. The interest of researchers is now moving towards the use of very dense point clouds to derive surface properties at desired spatial scales. However, a limited number of papers and experiences have focused on time-related issues, monitoring, dynamic assessments, quasi-real-time investigations of phenomena from repeated surveys, and automated processing. This still represents a challenging approach to surveying. This Special Issue aims at papers related to the study of novelties and advances in these key areas of photogrammetry and remote sensing, placing emphasis on new algorithms, modern and/or forthcoming sensors, improvements in data fusion and processing strategies, as well as the assessment of their reliability with the careful consideration of quality assurance/quality control and error budget. Additionally, this Special Issue aimed to collect papers devoted to the application of such innovations as proof of the contribution offered in the observation of the natural and built environment and our understanding of phenomena at required spatial scale.

After the revision procedure, five papers focused on satellite and aerial/terrestrial images and videos processing were selected and published. Three of them referred to innovative applications from satellite platform. In particular, Agapiou [1] used backscattered signals and interferometric synthetic aperture radar (InSAR) analysis from the Sentinel-1 radar in addition to medium-resolution Sentinel-2 optical data images and a cloud-based facility to produce a damage proxy map of the Beirut explosion which occurred on August 4, 2020. Qiao et al. [2] presented a novel method for detecting changes after a natural disaster using an optical flow-based method and adaptive thresholding segmentation able to detect pixel-based motion tracking at fast speed from video sequences composed of successive passages of high-resolution satellites. Papers based on satellite data include the work by Loghin et al. [3]. These authors discuss the use of high-resolution stereo and multi-view imagery for digital surface model (DSM) derivation over large areas for numerous applications in topography, cartography, geomorphology, and 3D surface modelling. In particular, the paper was focused on applications based on Pléiades (0.70 m) and WorldView-3 (0.31 m) imagery for the reconstruction of small isolated objects with the assessment of their detectability, by estimating heights as a function of object type and size, and the successive validation of measurements. Two papers focused on photogrammetric data processing are included in this Special Issue. The paper by Nocerino et al. [4] evaluated whether the integration of visibility information (image orientation) and photo-consistency could potentially lead to an improvement of the mesh quality (and successive products) with tests carried out on diverse datasets. Metrics were introduced and considered to present qualitative and quantitative assessments of the results. Finally, Nikolov and Madsen [5] introduced a metric for noise estimation in highly detailed 3D SfM reconstructions. In particular, the authors discussed a possible approach to distinguishing real surface roughness from reconstruction noise and proposed a number of geometrical and statistical metrics for noise assessment based on both the reconstructed object and the capturing camera setup.

We hope that the scientific community involved in photogrammetry and remote sensing will find the papers published in this Special Issue useful for their future investigations.
